# Pretargeted immuno-PET of CEA-expressing intraperitoneal human colonic tumor xenografts: a new sensitive detection method

**DOI:** 10.1186/2191-219X-2-5

**Published:** 2012-01-27

**Authors:** Rafke Schoffelen, Winette TA van der Graaf, Robert M Sharkey, Gerben M Franssen, William J McBride, Chien-Hsing Chang, Peter Laverman, David M Goldenberg, Wim JG Oyen, Otto C Boerman

**Affiliations:** 1 Dept. of Nuclear Medicine, Radboud University Nijmegen Medical Centre, 6500 HB, Nijmegen, 9101, The Netherlands; 2 Dept. of Medical Oncology, Radboud University Nijmegen Medical Centre, Nijmegen, 6500 HB, Nijmegen, 9101, The Netherlands; 3 Garden State Cancer Center, Belleville, NJ, 07109, USA; 4 Immunomedics, Inc., Morris Plains, NJ, 07950, USA; 5 IBC Pharmaceuticals, Immunomedics, Inc., Morris Plains, NJ, 07950, USA

**Keywords:** colorectal cancer, carcinoembryonic antigen, imaging, PET, pretargeting, bispecific antibodies

## Abstract

**Background:**

In this study, pretargeted immuno-positron-emission tomography [PET] with a bispecific monoclonal anti-carcinoembryonic antigen [CEA] (CEACAM5) × anti-hapten antibody (bispecific monoclonal antibody [bsmAb]) and a small (1.5 kD) peptide labeled with ^68^Ga was compared to fludeoxyglucose [^18^F-FDG]-PET for detecting intraperitoneal [i.p.] CEA-expressing human colonic tumor xenografts in nude mice.

**Methods:**

Two groups of female BALB/c nude mice were inoculated with LS174T human colonic tumor cells i.p. One group received 5 MBq ^18^F-FDG, and the other received intravenous injections of the bsmAb, followed 16 h later with 5 MBq of ^68^Ga-labeled peptide. One hour after the radiolabeled peptide or FDG was given, micro-PET/computed tomography images were acquired. Thereafter, the uptake of the ^68^Ga or ^18^F in dissected tissue was determined.

**Results:**

Within 1 h, high uptake of the ^68^Ga-labeled peptide in the tumor lesions (23.4 ± 7.2% ID/g) and low background activity levels were observed (e.g., tumor-to-intestine ratio, 58 ± 22). This resulted in a clear visualization of all intra-abdominal tumor lesions ≥ 10 μL and even some tumors as small as 5 μL (2 mm diameter). ^18^F-FDG efficiently localized in the tumors (8.7 ± 3.1% ID/g) but also showed physiological uptake in various normal tissues (e.g., tumor-to-intestine ratio, 3.9 ± 1.1).

**Conclusions:**

Pretargeted immuno-PET with bsmAb and a ^68^Ga-labeled peptide could be a very sensitive imaging method for imaging colonic cancer, disclosing occult lesions.

## Background

Colorectal cancer is a frequently diagnosed cancer type. It is the third most common cancer in both men and women in the Western world [1,2]. The overall 5-year survival is 40% to 60% [3,4]. The prognosis is mainly determined by the presence of local or distant metastases, especially in the liver and peritoneum, which occur in half of the patients. Only patients with a limited number of liver or lung metastases have a chance for cure by extensive surgery, generally combined with chemotherapy. However, up to half of the patients selected for metastasectomy have inoperable disease at laparotomy [5]. Therefore, preoperative staging for detecting extrahepatic disease is crucial to avoid futile major surgery [6].

Specific detection of malignant colorectal tumor lesions could be achieved by (pretargeted) antibody-guided radionuclide imaging. The combination of the specificity of antibody targeting and the sensitivity of positron-emission tomography [PET] is very promising. Radiolabeled antibodies have been tested for the detection of several cancer types. However, imaging with radiolabeled whole antibodies requires a relatively long interval between injection and imaging acquisition for adequate contrast to develop due to the slow accretion of intact antibodies in tumors and their slow clearance [7]. Pretargeting techniques were developed to improve radioimmunotargeting of tumors [8]. A two-step pretargeting method using bispecific monoclonal antibodies [bsmAb] has been developed. First, an unlabeled bsmAb with affinity for both the tumor and a small radiolabeled molecule is injected. When the bsmAb has cleared from the blood and has accumulated in the tumor, a radiolabeled and hapten-conjugated peptide that clears rapidly from the blood and the body but is trapped in the tumor by the anti-hapten binding arm of the bsmAb is administered [9-11]. Such a pretargeting method allows imaging within 1 h after the injection of the radiolabeled peptide, with high contrast, in animal models.

Coupling two haptens together improves peptide uptake and stability by a process known as affinity enhancement [12]. Chelate-metal complexes, such as DTPA-In, have been used as haptens [13].

Fludeoxyglucose [FDG]-PET/computed tomography [CT] has an established role in the work-up of patients with metastasized colorectal cancer and could change patient management in > 25% of patients [14-16]. Other clinical indications for PET scanning in patients with colorectal cancer are the detection of disease recurrence and characterization of undefined lesions on conventional imaging [17-20]. However, since FDG is a nonspecific tracer, it also has uptake in other tissues (e.g., physiological uptake in the bowel and uptake in (postsurgical) inflammatory or infectious lesions). FDG-PET frequently causes diagnostic dilemmas in assessing peritoneal disease [21-24].

In the present study, we examined the sensitivity of pretargeting with a bispecific monoclonal anti-carcinoembryonic antigen [CEA] × antihistamine-succinyl-glycine [HSG] antibody, TF2, and a ^68^Ga-labeled peptide, IMP288. Pretargeted immuno-PET was compared to ^18^F-FDG-PET in a preclinical orthotopic model in mice with small, intraperitoneally growing CEA-expressing colonic tumor lesions.

## Methods

### Pretargeting reagents TF2 and IMP288

The bsmAb, TF2, and the peptide IMP288 were provided by Immunomedics (Morris Plains, NJ, USA). The preparation of TF2 and binding properties has previously been described [25-29]. Gel filtration chromatography showed that TF2 bound > 90% of ^68^Ga-IMP288 peptide. IMP288 was synthesized and purified as described by McBride et al. [30]. IMP288 is a DOTA-conjugated D-Tyr-D-Lys-D-Glu-D-Lys tetrapeptide in which both lysine residues are substituted with an HSG moiety via their ε-amino group: 7,10-tetraazacyclododecane-*N*, *N*',*N*″,*N*″'-tetraacetic acid [DOTA]-D-Tyr-D-Lys(HSG)-D-Glu-D-Lys(HSG)-NH_2_.

TF2 was labeled with ^125^I (PerkinElmer, Waltham, MA, USA) by the iodogen method as described previously [31] to a specific activity of 58 MBq/nmol. ^125^I-labeled TF2 was purified by eluting the reaction mixture with phosphate-buffered saline [PBS] and 0.5% *w*/*v *bovine serum albumin [BSA] (Sigma Chemicals, Sigma-Aldrich Corporation, St. Louis, MO, USA) on a PD-10 column (GE Healthcare Bio-Sciences AB, Uppsala, Sweden). IMP288 was labeled with ^68^Ga as described previously [32]. Radiolabeling and purification for administration could be accomplished within 45 min. The final product was adjusted to have a specific activity of 20 MBq/nmol at the moment of injection. ^18^F-FDG was obtained from B.V. Cyclotron VU, Amsterdam, The Netherlands.

### Quality control of the radiolabeled preparations

Radiochemical purity of the radiolabeled TF2 and IMP288 preparations was determined as described previously [32]. In all experiments, the radiochemical purity of ^125^I-TF2 and ^68^Ga-IMP288 preparations exceeded 95%.

### Animal experiments

All studies were approved by the Institutional Animal Welfare Committee of the Radboud University Nijmegen Medical Centre and conducted in accordance with their guidelines (revised Dutch Act on Animal Experimentation, 1997). Animals were accustomed to laboratory conditions for 1 week before use and housed in individually ventilated isolator cages under standard laboratory conditions (temperature, 20°C to 24°C; relative humidity, 50% to 60%; and light-dark cycle, 12 h) with free access to animal chow and water.

Female nude BALB/c mice (6 to 8 weeks old), weighing 20 to 25 g, received an intraperitoneal injection of 0.5 mL of a suspension of 1 × 10^6 ^LS174T cells, a CEA-expressing human colon carcinoma cell line (CCL-188; passage 7; American Type Culture Collection, Manassas, VA, USA). Three weeks after tumor cell inoculation, one group of five mice was injected intravenously with 5.0 nmol TF2 (0.2 mL) labeled with a trace amount of ^125^I (0.4 MBq). Sixteen hours later, ^68^Ga-IMP288 (5 MBq/025 nmol) was administered intravenously in 0.2 mL as described previously [32]. The other group of five mice received 5 MBq ^18^F-FDG intravenously [i.v.]. The mice were fasted for 10 h before the ^18^F-FDG injection, anesthetized, and kept warm at 37°C. The mice were euthanized 1 h after the injection of ^68^Ga-IMP288 or ^18^F-FDG by CO_2_/O_2 _asphyxiation, followed by cardiac puncture to obtain blood.

PET/CT scans of the mice were acquired 1 h after the injection of ^68^Ga-IMP288 or ^18^F-FDG with an Inveon animal PET/CT scanner (Siemens Preclinical Solutions, Erlangen, Germany) having an intrinsic spatial resolution of 1.5 mm [33]. The animals were placed in a supine position. PET scans were acquired for 15 min, preceded by CT scans for anatomical reference (spatial resolution, 113 μm; 80 kV; 500 μA; exposure time, 300 ms). Scans were reconstructed using Inveon Acquisition Workplace software (version 1.5; Siemens Preclinical Solutions) using a three-dimensional ordered subset expectation maximization/maximum *a posteriori *algorithm with the following parameters: matrix, 256 × 256 × 159; pixel size, 0.43 × 0.43 × 0.8 mm^3^; and maximum *a posteriori *prior β 0.5.

After the scans, the mice were dissected, and the abdomen was systematically and meticulously examined for tumors. The location of each lesion was documented, weighed, and measured, and then the activity in each lesion was determined in a gamma counter. The other organs of interest were weighed and counted in a gamma counter with standards prepared from the injected products, using appropriate energy windows for the radionuclide of interest. The percentage of the injected dose per gram tissue [% ID/g] was calculated. The correlation between the weight and uptake of ^125^I-TF2 as ^68^Ga-IMP288 per lesion was calculated.

Immunohistochemical analysis of CEA was performed on 4-μm-thick formalin-fixed, paraffin-embedded tissue sections. The sections were deparaffinized in xylol and rehydrated through a graded ethanol into water series. To block endogenous peroxidase, slides were blocked with 3% hydrogen peroxide in phosphate buffered saline (10 min at room temperature). Then sections were blocked with 20% normal goat serum (Vector Laboratories Inc., Burlingame, USA) in 1% BSA-PBS (30 min at room temperature [RT]). Subsequently, tumor sections were incubated with a 1:12,000 dilution of polyclonal rabbit anti-CEA antibody (A0115, Dako, Glostrup, Denmark) overnight at 4°C, followed by incubation with a goat-anti-rabbit biotinylated secondary antibody (1/200 in 1% BSA-PBS) (Vector Laboratories Inc., Burlingame, CA, USA) for 30 min at RT. Finally, avidin-biotin-enzyme complex (Vector Laboratories Inc.) was applied for 30 min at 37°C, and 3,39-diaminobenzidine was used to develop the tumor sections. Human colon carcinoma was used as a positive control, and substitution of the primary antibody with 1% BSA-PBS was used as the negative control.

### Analysis of the PET images

PET/CT images were scored by a blinded, independent, experienced nuclear physician (W.O.), being asked to record the presence of intra-abdominal tumor lesions. When lesions were present, he was asked to draw a region of interest [ROI] around the tumor. Each lesion was given a number on a 1 to 3 scale that defined the reader's confidence that the uptake was related to a tumor (definitely, probably, or possibly a tumor). The imaging findings were then compared with the tumor lesions found at dissection. The detection rates for tumors < 10 μL and ≥ 10 μL were calculated, corresponding with a sphere diameter of < 2.7 or ≥ 2.7 mm, respectively.

### Statistical analyses

Statistical analysis was performed using the SPSS software (Chicago, IL, USA) and GraphPad Prism version 5.00 for Windows (GraphPad Software, San Diego, CA, USA). Means and standard deviations were used to describe continuous data, unless stated otherwise. Correlations were determined using a Spearman's correlation test. The level of significance was set at *p *< 0.05.

## Results

### Tumor growth

Three weeks after the intraperitoneal injection of the LS174T cells, the mice did not show clinical signs of discomfort or change in body weight. At dissection, the abdomen contained multiple solid tumor lesions (median, *n *= 10/mouse; range, 4 to 17). Most frequent localizations were at the rectovesical pouch, the mesentery, and the subhepatic, -splenic, and -phrenic spaces. Some tumor nodules were adjoining in groups of two or three lesions. Three-dimensional caliper measurements indicated that the maximum diameter of the tumor lesions varied between 1 and 15 mm (median, 5 mm), and weights varied between 0.3 and 650 mg (median, 16 mg).

### Biodistribution

**Figure 1:**
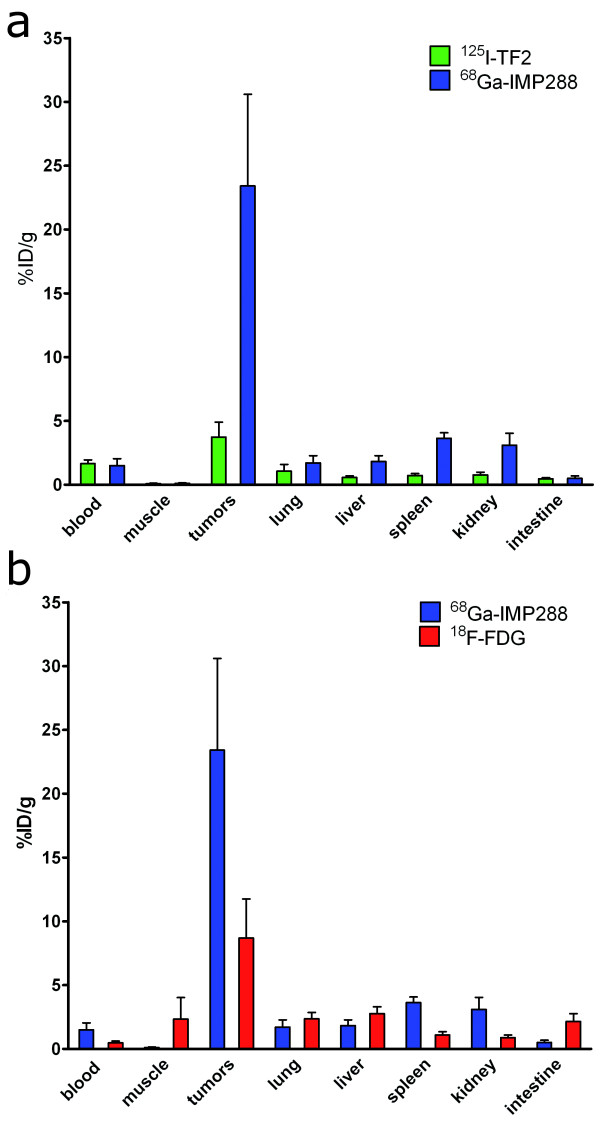
**Biodistribution**. (**a**) 6.0 nmol ^125^I-TF2 (0.37 MBq) and 0.25 nmol ^68^Ga-IMP288 (5 MBq) 1 h after i.v. injection of ^68^Ga-IMP288 in BALB/c nude mice with intraperitoneal CEA-expressing LS174T tumors. (**b**) 0.25 nmol ^68^Ga-IMP288 (5 MBq) and ^18^F-FDG (5 MBq) 1 h after i.v. injection in the BALB/c nude mice with intraperitoneal CEA-expressing LS174T tumors. Values are given as means ± standard deviation (*n *= 5).

The biodistribution of ^125^I-TF2 and ^68^Ga-IMP288 in the mice is shown in Figure 1a. High uptake of the bsmAb (3.73 ± 1.2% ID/g) and peptide (23.4 ± 7.2% ID/g) in the tumor lesions was observed with very low accretion in the normal organs. This resulted in high tumor-to-normal-tissue ratios of ^68^Ga-IMP288 (e.g., tumor-to-intestine ratio, 58 ± 22; tumor-to-liver ratio, 15 ± 3).

**Figure 2:**
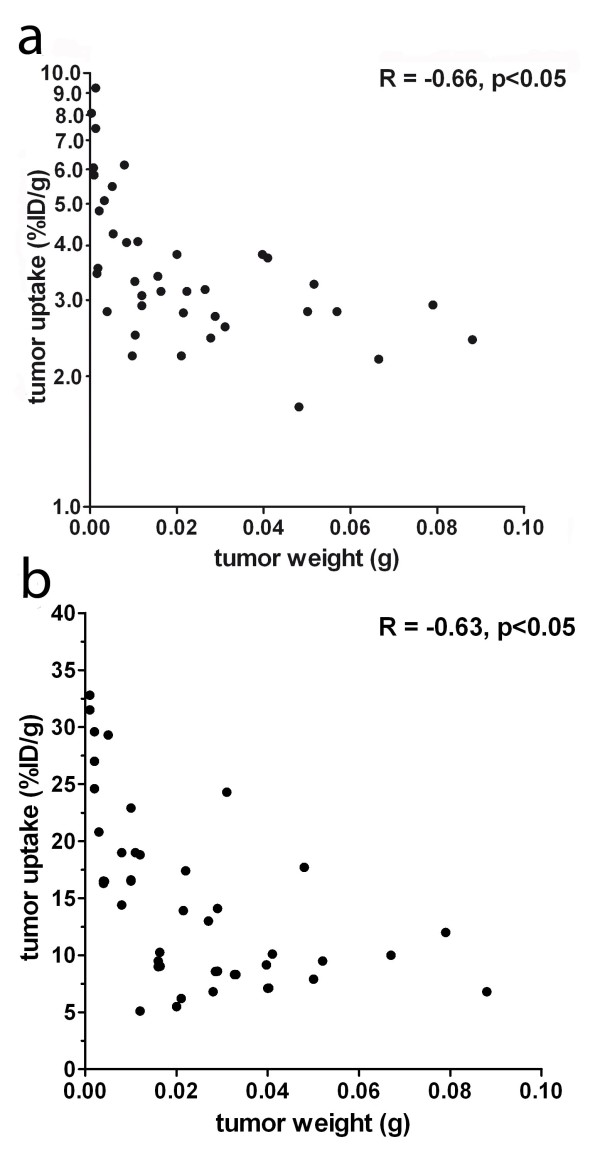
**Correlation between tumor uptake of ^125^I-TF2 (A) and ^68^Ga-IMP288 (B) and tumor size**. (Spearman's rho = -0.66, *p *< 0.05 and Spearman's rho = -0.63, *p *< 0.05, respectively.)

^18^F-FDG localized efficiently in the tumors (8.7 ± 3.1% ID/g; Figure 1b) but with physiological uptake in various normal tissues and with lower tumor-to-normal tissue ratios (e.g., tumor-to-intestine ratio, 3.9 ± 1.1; tumor-to-liver ratio, 2.9 ± 0.5). Tumor uptake of both ^125^I-TF2 and ^68^Ga-IMP288 correlated inversely with tumor size, as shown in Figure 2a, b (Spearman's rho = -0.66, *p *< 0.05, and Spearman's rho = -0.63, *p *< 0.05, respectively).

### PET/CT images

**Figure 3:**
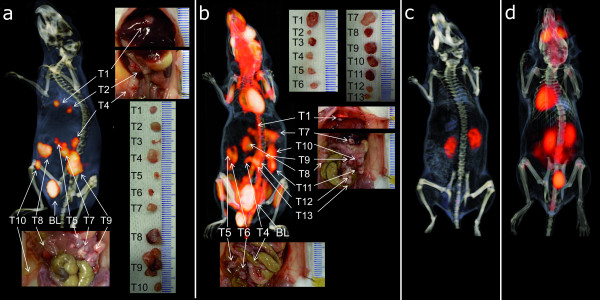
**Images**. 3D-volume rendering of the pretargeted immuno-PET scan (**a**) and the FDG-PET/CT scan (**b**) of the BALB/c nude mice with intraperitoneal LS174T tumors that received 6.0 nmol TF2 and 5 MBq ^68^Ga-IMP288 (0.25 nmol) with a 16-h interval (a) or ^18^F-FDG (b). The animals were imaged 1 h after ^68^Ga-IMP288 or ^18^F-FDG injection. Digital pictures were made during dissection to localize and measure individual tumors. On the pretargeted immuno-PET/CT images (a), all dissected tumors were very clearly distinguishable, except for the two very small tumors (1.2 and 4.7 μL, respectively). In the FDG-PET/CT images (b), arrows are pointed at the localizations where tumors were found at dissection, but the signal was difficult to be discriminated from the intestines. Figure 3**c**, **d **shows the PET/CT images of mice without intraperitoneal images after TF2 and ^68^Ga-IMP288 injection (c) or ^18^F-FDG injection (d).

Immuno-PET with TF2 and ^68^Ga-IMP288 resulted in a clear delineation of the tumors. An example of a PET/CT image is shown in Figure 3a. It shows the cross sections through several tumor lesions. The photographs show their localization in the abdomen as well as their size. Apart from the activity in the bladder, very low uptake in normal tissues was seen. Due to the highly specific uptake in the tumor lesions and low background concentration, the immuno-PET/CT images could even be used to guide the localization of tumor lesions during dissection. Tumors that were more difficult to find macroscopically because they were localized in the retroperitoneal cavities or posterior to the liver were easily seen and localized on the images.

**Figure 4:**
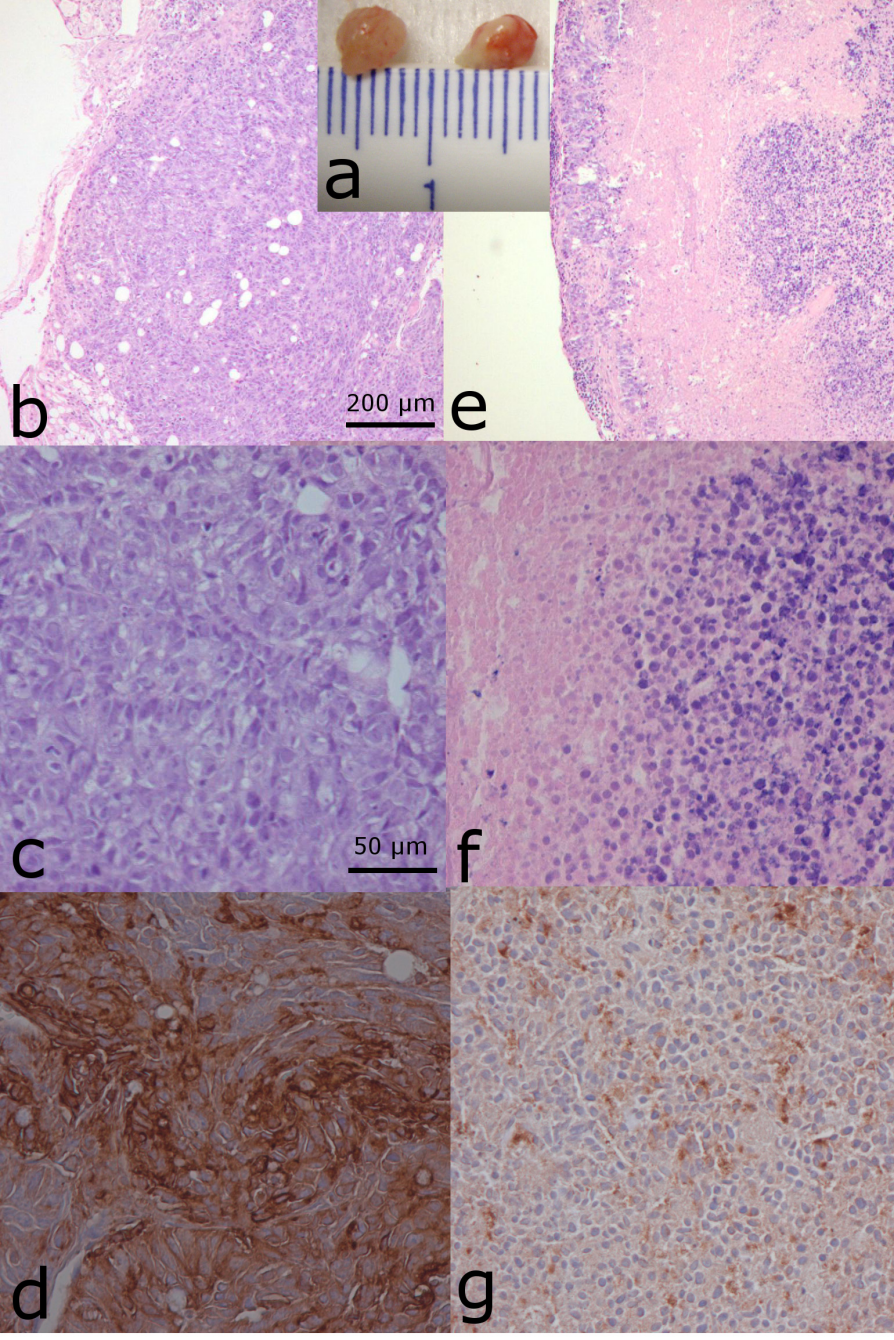
**Immunohistochemistry**. Two tumor lesions dissected from the abdomen of a BALB/c nude mouse that received ^68^Ga-IMP288 after pretargeting with TF2 (**a**). One lesion (a: left lesion) showed normal vital tumor cells on microscopic hematoxylin and eosin [HE]- and CEA-stained images (**b**: HE, × 5; **c**: HE, × 20; and **d**: CEA, × 20) and high specific tumor uptake of ^68^Ga-IMP288 (17.7% ID/g). On the contrary, another lesion in the same animal (a: right lesion) showed much lower tumor activity concentration (0.49% ID/g) in biodistribution and a much lower signal on the PET/CT images. This result was explained by the HE sections and CEA-stained images showing > 90% of non-vital tumor tissue (necrosis and infiltrated lymphocytes), lacking CEA expression (**e**: HE, × 5; **f**: HE, × 20; and **g**: CEA, × 20).

Interestingly, one lesion that was macroscopically doubtful to be a tumor, and showing minimal uptake on immuno-PET, had an activity concentration as low as 0.49% ID/g. This uptake level was much lower than that of the other lesions in the same animal (range, 16.3 to 29.6% ID/g). This lesion with the low uptake was shown by immunohistochemistry to consist > 90% of necrotic tissue and infiltrated leukocytes, lack CEA expression, and have only a small rim of vital tumor cells (Figure 4), which explains its low signal on immuno-PET.

In contrast, it was more difficult to discriminate the tumor lesions from other intra-abdominal structures on the FDG-PET images because the uptake in the tumors was only slightly higher than that in the intestines, as is shown in Figure 3b. FDG-PET images showed physiological uptake in the brain and the myocardium.

To illustrate the low uptake of the pretargeting peptide in the background, the immuno-PET/CT and FDG-PET/CT images of the mice without intraperitoneal tumors, which were imaged according to the same scanning protocol, are shown in Figure 3c, d. In pretargeted immuno-PET/CT images, only a low signal in the kidneys was observed, whereas no uptake was observed in the other normal organs. The FDG-PET/CT image of the animal without abdominal tumors clearly showed uptake in the bowel.

### Sensitivity

There was a major difference in the number of detected lesions in the immuno-PET/CT compared with the FDG-PET/CT. Table 1 shows the number of tumors that were correctly aligned by the independent nuclear physician for each imaging method. For the pretargeted immuno-PET, all tumor lesions ≥ 10 μL were detected (100%, 23/23). A separate analysis for the smaller lesions, < 10 μL, showed a detection rate of 20% (3/15). The score on the probability scale was 'definitely positive' for 88% of the delineated lesions. In contrast, in the FDG-PET images, the detection rate of the tumors ≥ 10 μL was only 48% (13/27). A similar small proportion of the smaller lesions were found by FDG-PET/CT compared to immuno-PET/CT (25%, 3/12). Interestingly, the nuclear medicine physician was much less confident about aligning the ROIs in the FDG-PET/CT images. For none of the lesions, he scored 'definitely positive' and only 'possibly positive' for 69% (11/16).

**Table 1:** Number of tumors correctly aligned by pretargeted immuno-PET/CT and FDG-PET/CT

		Pretargeted immuno-PET/CT	FDG-PET/CT
Tumors > 10 μL	Dissected	23	27
	Detected in images	23 (100%)	13 (48%)
Tumors < 10 μL	Dissected	15	12
	Detected in images	3 (20%)	3 (25%)
Probability assigned by the nuclear physician	Definitely positive	23 (88%)	0
	Possibly positive	3 (12%)	11 (69%)
	Probably positive	0	5 (31%)

Alignment and confidence rate was done by the independent nuclear physician.

## Discussion

This study showed that pretargeted immuno-PET is a very sensitive imaging modality to detect CEA-expressing tumor lesions in an orthotopic mouse model. The intraperitoneal tumors were clearly delineated with a high tumor-to-background contrast, providing high sensitivity: all tumor lesions ≥ 10 μL were detected with this method at a very good confidence rate. The smallest lesions that were detected had a volume as low as 5 to 8 μL, which is in the same range of the spatial resolution of the dedicated animal PET scanner.

The animal model used in this study was well characterized by Koppe et al [34]. The human colon carcinoma cell line LS174T has a reproducible growth pattern in BALB/c nude mice after intraperitoneal injection. Three weeks after tumor cell inoculation, small tumor nodules were observed in the rectovesical pouch, the mesentery, and the subhepatic, -splenic, and -phrenic spaces. The preclinical model mimics peritoneal disease of patients with metastasized colorectal cancer [35,36].

In a previous imaging study, we demonstrated the feasibility of pretargeted immuno-PET using ^68^Ga- or ^18^F-labeled di-HSG peptides in mice with subcutaneous tumors [32]. In the current study, the activity concentration of the ^68^Ga-labeled IMP288 in the intraperitoneal tumors was similar to that in the subcutaneous tumors [32,37]. In our intraperitoneal tumor model, the variation in tumor size was much wider than that in the subcutaneous model. Our biodistribution results showed an inverse relationship between tumor weight and activity concentration. This correlation corresponds with the findings of other investigators [38-41]. Sharkey et al. showed specific uptake of ^124^I-labeled peptide after pretargeting with TF2 in microdisseminated human colon cancer colonies in the lungs of nude mice. In that model, high tumor-to-non-tumor ratios were obtained, illustrating the excellent tumor targeting potential of the pretargeting strategy [42].

FDG-PET/CT has shown high sensitivity and negative predictive value in diagnosing CRC [43,44]. Therefore, it was used in the present study as a reference method. The imaging quality of FDG-PET in this preclinical study was optimized by minimizing uptake of FDG in other organs by anesthesia, fasting, and warming of the animals [45]. Its uptake in the myocardium, brain, intestines, and liver is comparable to the clinical situation. The ratios between normal and tumor tissues might have appeared to be less favorable than in patients, which might have compromised the detection of the tumors.

Based on our preclinical results, we feel that pretargeted immuno-PET can be of additive value in the clinical setting. When staging patients with primary tumors in the detection of eventual metastases, a highly sensitive and specific imaging method is required. Furthermore, in patients to be screened prior to curative liver metastasectomy, the disclosure of occult extrahepatic lesions will prevent useless operations. More so, immuno-PET can help select patients who could undergo radioimmunotherapy. As the pretargeting system with the DOTA-conjugated peptides is very flexible, it can be labeled with a broad variety of radionuclides, such as ^90^Y and ^177^Lu for pretargeted radioimmunotherapy, or with ^111^In and ^99 m^Tc for SPECT imaging. Our preclinical results show similar biodistribution of the ^111^In/^177^Lu- or ^68^Ga-labeled peptide [37]. Images about targeting known, non-biopsied lesions can confirm antigen expression and accessibility of the therapeutic dose. Information on the biodistribution and pharmacokinetics can help adjust treatment regimes by providing dosimetry data. This could be used to optimize dosing and to avoid toxicities.

For clinical application, ^68^Ga has some major advantages. It is readily available in a nearly carrier-free state from an in-house ^68^Ge/^68^Ga generator. IMP288-DOTA can be stably and rapidly labeled with ^68^Ga. Its half-life matches the pharmacokinetics of the peptide. In the present study, the positron range of ^68^Ga (median range, 3.5 mm) might have limited image resolution. Visser et al. [33] showed that with the intrinsic spatial resolution (approximately 1.5 mm) of our state-of-the-art, small-animal PET scanner, the finite positron range has become the limiting factor for the overall spatial resolution and activity recovery in small structures imaged with ^68^Ga. Combined with the partial volume effect, this could explain the lower detection rate of the smallest tumor lesions with pretargeted immuno-PET despite the higher radioactivity concentration of TF2 and ^68^Ga-IMP288 in the smaller tumors.

Due to the flexibility of the di-HSG peptides, the use of other PET radionuclides for this pretargeting system can be explored. ^18^F, the most widely used positron-emitting radioisotope, would be suitable due to its short positron range in the tissue (0.62 mm), which might increase the image resolution. McBride and, subsequently, Laverman et al. developed an innovative and rapid method for labeling peptides with ^18^F based on a metal chelator [46,47]. The biodistribution and PET images in the subcutaneous LS174T tumors in the nude mice showed the feasibility of this approach [32]. Translation of this preclinical imaging method to the clinical situation will show the effect of the intrinsic resolution of the clinical PET scanner in combination with the spatial resolution of the radionuclide.

## Conclusions

In summary, this study indicates that pretargeted immuno-PET with TF2 and ^68^Ga-IMP288 is a specific and sensitive method for detecting colon cancer in a preclinical model. Further clinical trials should focus on the diagnostic accuracy of pretargeted immuno-PET and determine its additional value in the clinical setting.

## Competing interests

WJM, DMG, and C-HC are employed by or have financial interest in Immunomedics, Inc. and/or IBC Pharmacauticals, Inc. The other authors declare that they have no competing interests.

## Authors' contributions

RS conceived the study, carried out the imaging studies and the analysis of the studies, and drafted the manuscript. WTAG conceived the study and helped draft the manuscript. RMS participated in the design of the study and helped draft the manuscript. GMF carried out the labeling procedures and the imaging studies. WJM and C-HC synthesized and purified the pretargeting agents. PL carried out the imaging studies and helped with the analysis of the studies. DMG participated in the design of the study and helped draft the manuscript. WJGO carried out the analysis of the imaging studies and helped draft the manuscript. OCB conceived the study, participated in the design of the study, and helped draft the manuscript. All authors read and approved the final manuscript.
